# Hantavirus Infection Is Inhibited by Griffithsin in Cell Culture

**DOI:** 10.3389/fcimb.2020.561502

**Published:** 2020-11-04

**Authors:** Punya Shrivastava-Ranjan, Michael K. Lo, Payel Chatterjee, Mike Flint, Stuart T. Nichol, Joel M. Montgomery, Barry R. O'Keefe, Christina F. Spiropoulou

**Affiliations:** ^1^Division of High Consequence Pathogens and Pathology, Viral Special Pathogens Branch, Centers for Disease Control and Prevention, Atlanta, GA, United States; ^2^Molecular Targets Program, Center for Cancer Research, National Cancer Institute, Frederick, MD, United States; ^3^Division of Cancer Treatment and Diagnosis, Natural Products Branch, Developmental Therapeutics Program, National Cancer Institute, Frederick, MD, United States

**Keywords:** hantaviridae, hantavirus, antiviral, haemorrhagic fever, griffithsin

## Abstract

Andes virus (ANDV) and Sin Nombre virus (SNV), highly pathogenic hantaviruses, cause hantavirus pulmonary syndrome in the Americas. Currently no therapeutics are approved for use against these infections. Griffithsin (GRFT) is a high-mannose oligosaccharide-binding lectin currently being evaluated in phase I clinical trials as a topical microbicide for the prevention of human immunodeficiency virus (HIV-1) infection (ClinicalTrials.gov Identifiers: NCT04032717, NCT02875119) and has shown broad-spectrum *in vivo* activity against other viruses, including severe acute respiratory syndrome coronavirus, hepatitis C virus, Japanese encephalitis virus, and Nipah virus. In this study, we evaluated the *in vitro* antiviral activity of GRFT and its synthetic trimeric tandemer 3mGRFT against ANDV and SNV. Our results demonstrate that GRFT is a potent inhibitor of ANDV infection. GRFT inhibited entry of pseudo-particles typed with ANDV envelope glycoprotein into host cells, suggesting that it inhibits viral envelope protein function during entry. 3mGRFT is more potent than GRFT against ANDV and SNV infection. Our results warrant the testing of GRFT and 3mGRFT against ANDV infection in animal models.

## Introduction

Andes virus (ANDV) and Sin Nombre virus (SNV) are zoonotic orthohantaviruses (referred here as hantaviruses) harbored by rodents and are responsible for nearly annual outbreaks of fatal human pulmonary syndrome disease in the Americas (Schmaljohn and Hjelle, 1997; Hooper et al., 2001; Jonsson et al., 2008; Brocato and Hooper, 2019). ANDV has case-fatality rates of 30–50% (Jonsson et al., 2010), and is the only hantavirus with documented evidence of person-to-person transmission (Martinez et al., 2005; Alonso et al., 2020). Although several therapeutics protect against lethal ANDV challenge in animals (Safronetz et al., 2013; Bird et al., 2016) none is currently approved for human use except for ribavirin, which is effective only when given early in the disease course (Chapman et al., 1999; Mertz et al., 2004). Inhibitors of virus entry often target protein interactions involved in receptor binding, but the glycosylation of the viral glycoproteins may represent another viable therapeutic target. Griffithsin (GRFT) is a homodimeric high-mannose oligosaccharide-binding lectin that specifically binds to *N*-linked high-mannose oligosaccharides present in viral envelope glycoproteins (Emau et al., 2007; Moulaei et al., 2015; Ishag et al., 2016). It has demonstrated broad-spectrum activity *in vitro* and *in vivo* against viruses including severe acute respiratory syndrome coronaviruses (O'Keefe et al., 2010), hepatitis C virus (Meuleman et al., 2011), Japanese encephalitis virus (Ishag et al., 2016), and Nipah virus (Lo et al., 2020), and currently is being evaluated in phase I clinical trials against human immunodeficiency virus 1 (HIV) (ClinicalTrials.gov Identifiers: NCT04032717, NCT02875119). Since hantavirus envelope glycoproteins Gn and Gc are heavily glycosylated (Shi et al., 2003), in this study, we assessed the *in vitro* antiviral activity of GRFT and its synthetic trimeric tandemer (3mGRFT) against ANDV and SNV.

## Methods

### Biosafety

All work with infectious virus was conducted in a biosafety level 3 (BSL-3) laboratory at the Centers for Disease Control and Prevention (CDC) following established BSL-3 standard operating procedures.

### Compounds, Cells, and Viruses

GRFT and its synthetic trimer tandemer 3mGRFT were expressed and purified as described previously (O'Keefe et al., 2009; Moulaei et al., 2015). African green monkey kidney (Vero-E6), human hepatoma (Huh7) and human fibrosarcoma (HT-1080) cells were cultivated as described previously (Moulaei et al., 2015; Shrivastava-Ranjan et al., 2016) Cells were incubated at 37°C in 5% CO_2_. ANDV (strain Chile 9717869) and SNV (strain MMR11) were propagated in Vero-E6 cells as described previously (Shrivastava-Ranjan et al., 2010).

### Immunofluorescence Image-Based Assay for Antiviral Activity

To test the inhibition of ANDV and SNV replication in cells pre-treated with GRFT or 3mGRFT, Vero-E6, Huh7 or HT-1080 cells were seeded at a density of 1 × 10^4^ /well of a 96-well plate the day prior to infection. Compounds were added to the cells, and 1 h later, the cells were infected with ANDV or SNV at the indicated multiplicity of infection (MOI). After indicated times post-infection, the monolayers were fixed with 10% formalin. The cells were then washed 3 times with phosphate buffered saline (PBS) and permeabilized with 0.1% (v/v) Triton X-100 in PBS for 10 min at room temperature, and nucleoproteins were detected with monoclonal antibody directed against the Puumala virus nucleoprotein; this antibody cross-reacts with against most known hantaviruses (1:10,000 antibody dilution in PBS supplemented with 2% bovine serum albumin). Primary antibodies were detected with goat-anti mouse Alexa 488 (1:500; Thermo Fisher). Cells were stained with CellMask Red and NucBlue (Life Technologies) and immunofluorescence microscopy was performed using the Operetta Imaging systems (PerkinElmer, Waltham, MA).

### Infectious Yield Assay

To assay effects of the compounds on viral titers, cell culture supernatants were harvested, and virus titrations were performed in Vero-E6 cells. Five days post infection, the cells were fixed, permeabilized, and stained to visualize viral proteins. End point viral titers were determined, and the 50% tissue culture infectious dose (TCID_50_) was calculated using the Reed and Muench method (Shrivastava-Ranjan et al., 2018).

### HIV Pseudo-Typed Entry Assays

HIV pseudo-typed particles bearing the glycoproteins of either ANDV, VSV or no glycoproteins were prepared and used as described previously (McNulty et al., 2013; Mohr et al., 2013). Briefly, LentiX-293T cells (Takara Bio, Mountain View, CA) were transfected with plasmids DNA encoding the HIV genome containing the firefly luciferase gene (pNL4-3.Luc.RE) and expression vectors encoding the viral glycoprotein or empty vector in a 1:8 ratio. Pseudo-typed viruses were quantitated by determining HIV matrix protein (p24) content. Viral glycoprotein-dependent entry assays were performed using HT-1080 cells and 6 ng of p24 pseudo-typed particles, with firefly luciferase expression detected using luciferase assay system (Promega, Madison, WI) 72 h post transduction.

### Cell Viability Assay

To measure potential cytotoxicity of GRFT and 3mGRFT, Vero-E6, Huh7 to HT-1080 cells were incubated with serial dilutions of either GRFT or 3mGRFT for 72 h. Cell viability was assayed using CellTiter-Glo assay reagent (Promega), with total luminescence measured using a Biotek HD1 synergy instrument. Luminescence levels (indicative of cellular ATP levels as a surrogate marker of cell viability) assayed in vehicle treated, uninfected cells were set as 100% viability.

### Statistical Analysis

Prism 7 (GraphPad) was used to generate graphs and perform statistical analysis. Statistical significance was calculated using the two-way ANOVA Tukey's multiple comparisons test, with α = 0.05.

## Results

### GRFT Inhibits ANDV Infection

**Figure 1:**
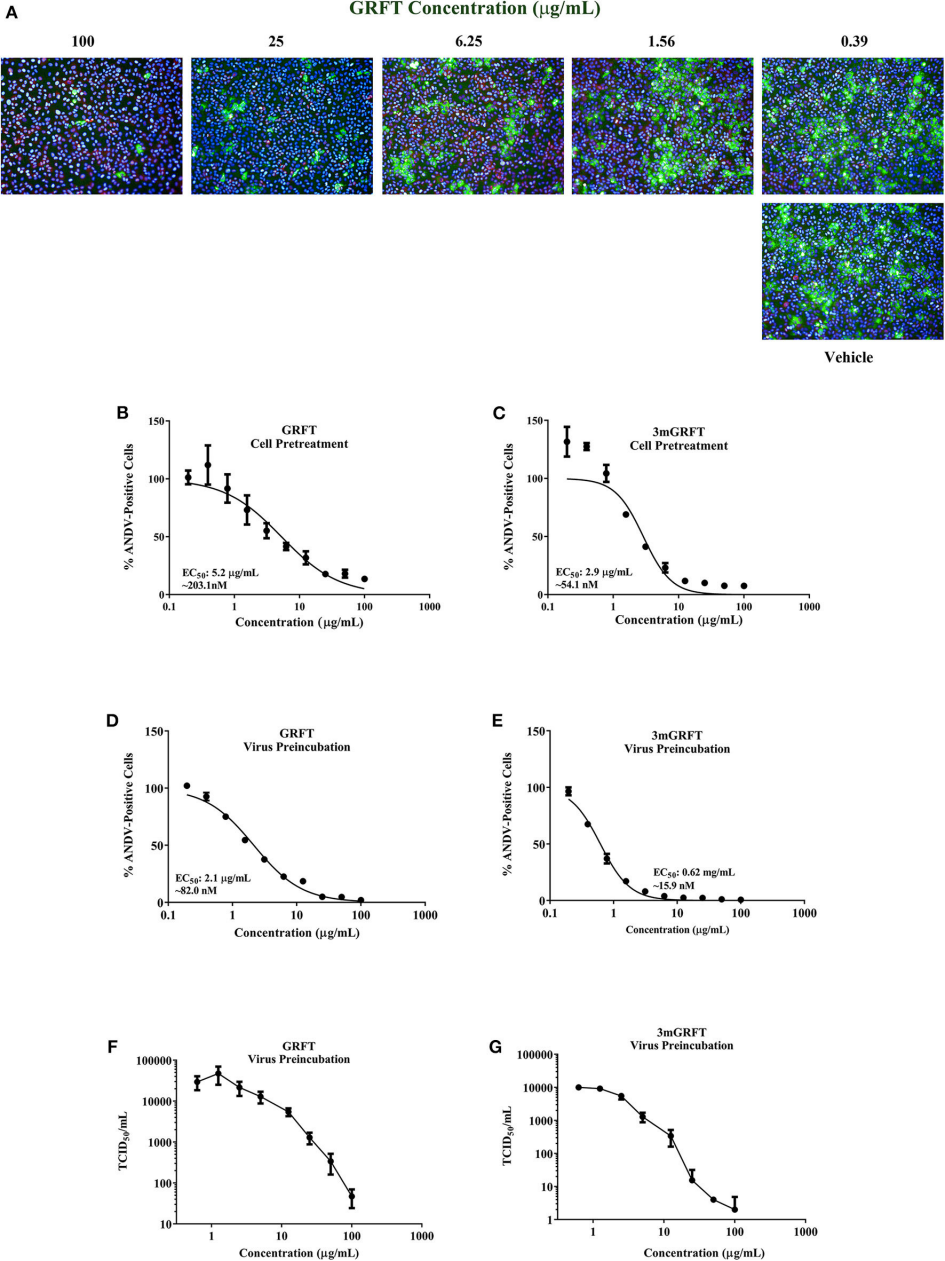
Griffithsin inhibits Andes virus replication. Griffithsin (GRFT) inhibited Andes virus (ANDV) replication in a concentration-dependent manner. **(A)** Vero-E6 cells were treated for 1 h with varying concentrations of GRFT before infection with ANDV at a multiplicity of infection (MOI) of 0.1. At 72 h post infection, the cells were fixed, permeabilized, and stained with an antibody against Puumala virus nucleoprotein that is cross-reactive with ANDV nucleoprotein. Green, ANDV nucleoprotein; blue, cell nuclei; red, cell cytoplasm. **(B,C)** Dose-response curve showing the quantitation of ANDV-infected cells after GRFT **(B)** or 3mGRFT **(C)** treatment (% normalized to the vehicle-only control). **(D,E)** Inhibition (neutralization) of ANDV by GRFT. Increasing concentrations of GRFT were pre-incubated with 2,000 TCID_50_ of ANDV at 37°C for 1 h, and virus-GRFT **(D)** or virus-3mGRFT **(E)** mixtures were added to Vero-E6 cells. After 2 h of incubation at 37°C, inoculum was removed, and cells were replenished with fresh medium. After 3 days, cells were fixed, imaged, and analyzed as in **(B)**. **(F,G)** GRFT inhibited titers of infectious ANDV. 2000 TCID_50_ of ANDV were pre-incubated with varying concentration of GRFT **(F)** or 3mGRFT **(G)** as in E. At 72 h, supernatants were harvested, and viral yield was determined by TCID_50_ assays on Vero-E6 cells. Graphs represent the mean ± SD and are representative of three independent experiments, performed in quadruplicate.

To determine whether GRFT could inhibit ANDV, Vero-E6 cells were pre-treated with varying concentrations of GRFT for 1 h before infection with ANDV at a MOI of 0.1. Seventy-two hours later, cells were fixed, permeabilized, and stained with an antibody raised against the nucleoprotein of Puumala virus that cross-reacts with nucleoproteins of other hantaviruses like ANDV and SNV. Stained cells were visualized using the Operetta imaging system, using nucleoprotein staining intensity as an indicator of viral replication (Mohr et al., 2013). Virus-specific staining was determined by measuring fluorescence at 488 nm, and acquired images were analyzed using Harmony software. GRFT concentrations that inhibited 50% of nucleoprotein intensity (50% effective dose [EC_50_]) were calculated from dose-response data fitted to a four-parameter logistic curve generated using GraphPad Prism 7 (GraphPad Software, La Jolla, CA, USA). GRFT inhibited wild-type ANDV in a dose-dependent manner, with EC_50_ of 5.2 μg/mL (203 nM) (Figure 1A) and showed minimal cytotoxicity in uninfected Vero-E6 cells [50% cytotoxic concentration [CC_50_] > 100 μg/mL or 3.9 μM] (Supplementary Figure 2).

GRFT is a domain-swapped homodimer with 3 identical oligosaccharide-binding domains on each monomer. The synthetically engineered trimeric tandemer 3mGRFT contains nine potential oligosaccharide binding sites and has been shown to be 10-fold more potent than native dimeric GRFT against HIV (Moulaei et al., 2015). We compared the antiviral activities of GRFT and 3mGRFT against ANDV and found 3mGRFT more than twice as potent (Figure 1B) as GRFT, with 3mGRFT EC_50_ of 2.1 μg/mL (54 nM) compared GRFT EC_50_ of 5.2 μg/mL (203 nM). 3mGRFT showed no detectable cytotoxicity at concentrations used in the experiment (CC_50_ > 100 μg/mL or > 2.8 μM; Supplementary Figure 2).

The antiviral effect of GRFT and 3mGRFT on ANDV infection was confirmed in two additional cell types; Huh7 and HT-1080. Pretreatment of Huh7 and HT-1080 cells with increasing concentrations of GRFT or 3mGRFT resulted in a dose-dependent inhibition of ANDV; EC_50_ values for GRFT was 180 and 184 nM, and for 3mGRFT was 62 and 75 nM, respectively (Supplementary Figure 1). No cytotoxicity was observed for GRFT and 3mGRFT in either cell type (GRFT: CC_50_ > 100 μg/mL or 3.9 μM; 3mGRFT CC_50_ > 100 μg/mL or > 2.8 μM, data not shown).

Next, we tested the effect of pre-incubating GRFT with ANDV prior to infection; ANDV was mixed with increasing concentrations of GRFT or 3mGRFT, and 1 h later, the ANDV-GRFT mixture was transferred onto Vero-E6 cells. Dose-response curves plotting percentages of ANDV-positive cells indicated that incubating ANDV with either GRFT or 3mGRFT prior to infection increased antiviral potency for both compounds; potency for GRFT increased 2.5-fold (EC_50_ of 82 nM; compare Figures 1E,B), while the potency of 3mGRFT increased 3.4-fold (EC_50_ of 15.9 nM; compare Figures 1F,D).

We then tested the effect of GRFT on yields of infectious ANDV 72 h post infection. Vero-E6 cells pre-incubated with serial dilutions of GRFT or 3mGRFT were infected with ANDV. After 2 h, the virus-drug mixture was removed, and fresh medium was added. Seventy-two hours post infection, the amount of secreted infectious particles present in the supernatant was determined by limiting dilution assays. A dose-dependent reduction in infectious virus titer was observed, with a > 2-log reduction in TCID_50_ of ANDV treated with GRFT (Figure 1F) and > 4 -log reduction in ANDV pre-treated with 3mGRFT (Figure 1G) compared to untreated controls.

### GRFT Inhibits ANDV Entry

**Figure 2:**
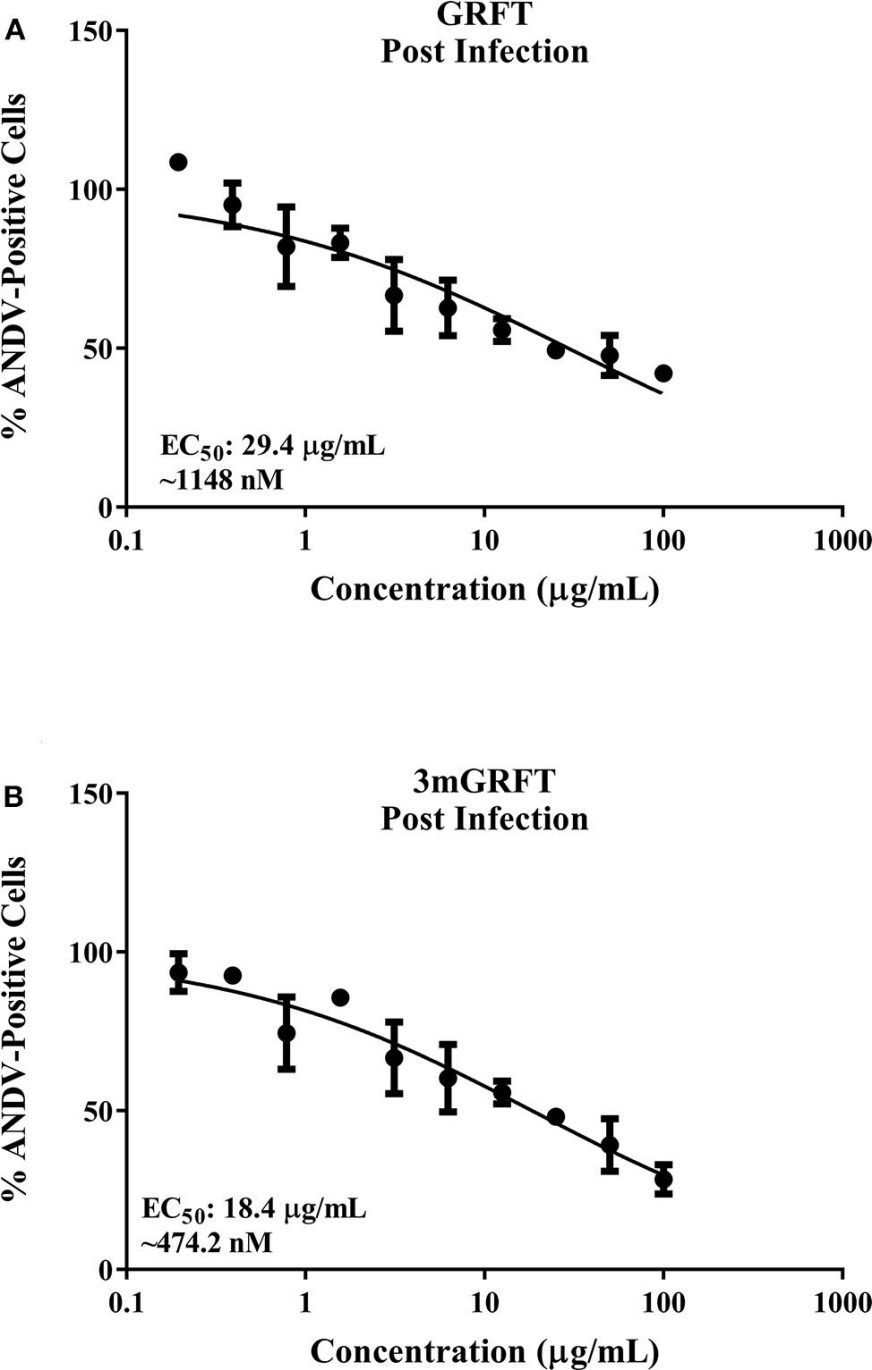
GRFT and 3mGRFT minimally affect post viral entry steps. Vero-E6 cells were infected with ANDV at MOI of 0.2 for 2 h at 37°C. Cells were then treated with varying concentrations of GRFT **(A)** or 3mGRFT **(B)**. At 72 h post infection, cells were fixed, stained, and analyzed as in Figure 1B. Dose-response curve shows quantitation of ANDV-infected cells treated with GRFT **(A)** or 3mGRFT **(B)** (% normalized to vehicle-only control). Graphs represent the mean ± SD and are representative of three independent experiments, performed in quadruplicate.

**Figure 3:**
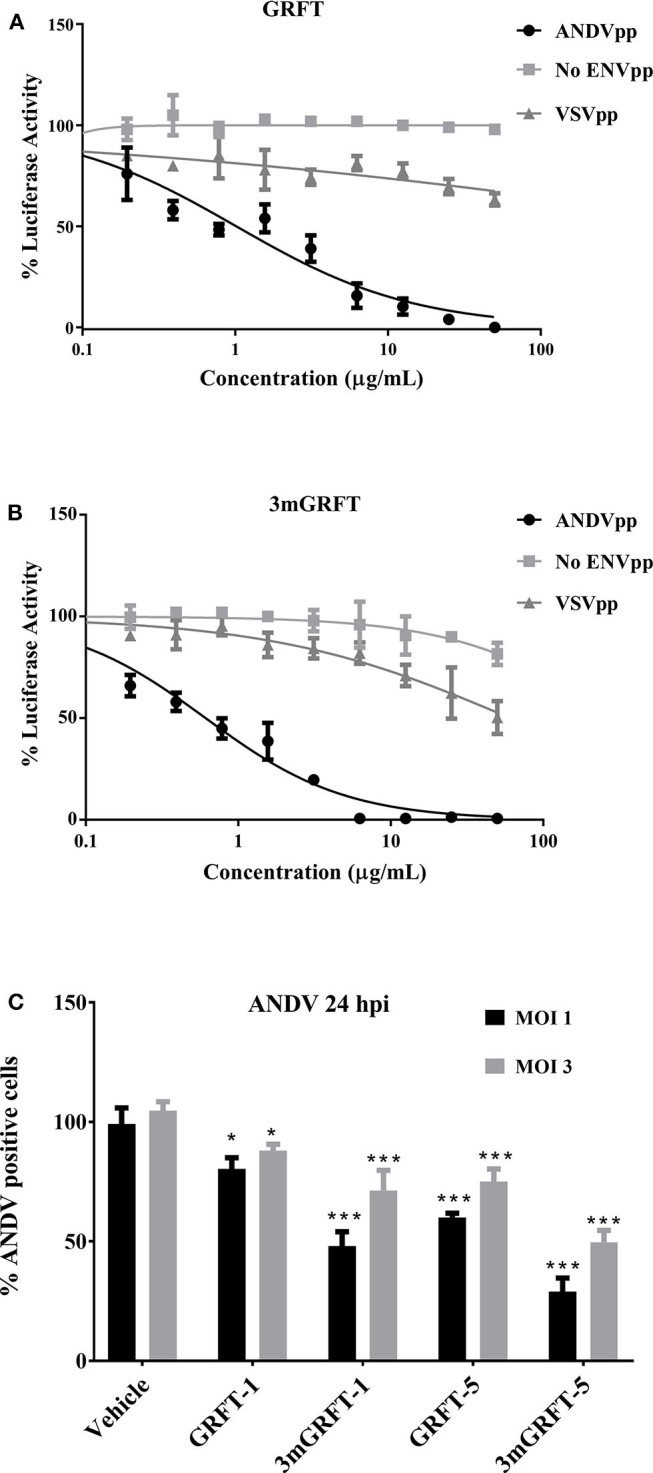
GRFT and 3mGRFT affect viral entry. HIV particles bearing glycoproteins of ANDV or vesicular stomatitis virus (VSV) were prepared and used as previously described. Viral glycoprotein-dependent entry assays were performed using HT1080 cells pre-treated with serial dilutions of GRFT and inoculated with either ANDV pseudo-particles (pp) or control VSVpp in the presence of GRFT **(A)** or 3mGRFT **(B)** at increasing concentrations. Intracellular firefly luciferase signal was measured 72 h post transduction and normalized to signal in untreated, untransduced cells. Graphs represent the mean ± SD and are representative of three independent experiments, performed in quadruplicate. **(C)** Vero-E6 cells were treated for 1 h with either 1 or 5 μg/mL of GRFT or 3mGRFT before infection with ANDV at MOI of either 1or 3. At 24 h post infection, the cells were fixed, permeabilized, and stained with an antibody against Puumala virus nucleoprotein as described in Figure 1. Column chart showing the quantitation of ANDV-infected cells treated with GRFT or 3mGRFT (% normalized to vehicle-only controls). Graphs represent the mean ± SD and are representative of 3 independent experiments, performed in quadruplicate. P^*^ < 0.001, P^***^ < 0.0001.

To evaluate whether GRFT treatment blocks ANDV entry, serial dilutions of GRFT or 3mGRFT were added to cells 2 h after ANDV infection. Dose-response curves indicated that post-infection treatment with GRFT or 3mGRFT only minimally inhibited ANDV infectivity, with approximately 5- and 8-fold increases in EC_50_ of GRFT (Figure 2A) and 3mGRFT (Figure 2B), respectively, compared to pre-treating virus 1 h before infection.

We then compared the ability of GRFT to block entry into cells of HIV-based pseudo-particles (pp) typed with ANDV or vesicular stomatitis virus (VSV) envelope glycoproteins. As shown in Figure 3A, GRFT dose-dependently inhibited cell entry of pp typed with ANDV but not VSV glycoprotein. 3mGRFT treatment similarly decreased entry of ANDVpp but not VSVpp (Figure 3B). As was observed with infectious virus, the antiviral potency of 3mGRFT (EC_50_ of 15.3 nM) was greater than that of GRFT (EC_50_ of 42.9 nM) (Figures 3A,B).

We further examined the antiviral effects of GRFT during viral entry by using authentic ANDV at high MOI. Vero-E6 cells were infected with at a MOI of 1 or 3 in the presence or absence of GRFT. As shown in Figure 3C, we observed reduced ANDV infection at both MOI for both compounds. However, the sensitivity of the virus to inhibition was dependent on the amount of virus in the inoculum. At a MOI of 1, as little as 1 μg/mL of 3mGRFT was enough to reduce ANDV infectivity by 50% in this assay (Figure 3C).

### GRFT Also Inhibits SNV Infection

**Figure 4:**
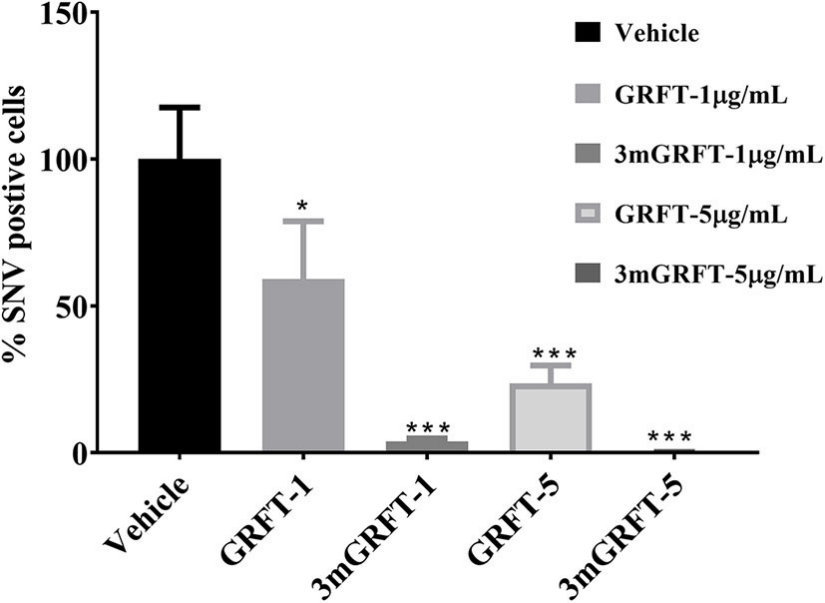
Antiviral effects of GRFT and 3mGRFT on Sin Nombre virus replication. Vero-E6 cells were treated for 1 h with either 1 or 5 μg/mL of GRFT or 3mGRFT before infection with Sin Nombre virus (SNV) at MOI of 0.2. At 72 h post infection, the cells were fixed, permeabilized, and stained with an antibody against Puumala virus nucleoprotein that cross-reacts with SNV nucleoprotein. Dose-response curve showing the quantitation of SNV-infected cells treated with GRFT or 3mGRFT (% normalized to vehicle-only controls). Graphs represent the mean ± SD and are representative of three independent experiments, performed in quadruplicate. P^*^ < 0.05, P^***^ < 0.001.

We then assessed the antiviral activity of GRFT and 3mGRFT against SNV, another hantavirus that causes pulmonary syndrome. Vero-E6 cells were pre-treated with GRFT or 3mGRFT before infection with SNV at a MOI of 0.2. Seventy-two hours post infection, cells were fixed and stained with anti-Puumala virus nucleoprotein monoclonal antibody as in Figure 1A. Both GRFT and 3mGRFT inhibited SNV replication more potently than they inhibited ANDV (Figure 4). While GRFT (5 μg/mL) treatment reduced infection by 75% (as determined by quantitating SNV-positive cells compared to untreated infected control), 3mGRFT (5 μg/mL) treatment reduced infection by 95%.

## Discussion

In summary, we utilized a cell-based fluorescent microcopy approach and virus titer reduction assays to demonstrate the *in vitro* activity of GRFT and its trimeric synthetic tandemer 3mGRFT against ANDV and SNV infection, confirming our initial hypothesis. The observed EC_50_ values of these compounds against ANDV were well within the range (nanomolar rather than picomolar) of those reported for other enveloped viruses (Meuleman et al., 2011; Ishag et al., 2016; Millet et al., 2016; Lo et al., 2020). Using both infectious virions and pseudo-particle systems, we clearly demonstrate that GRFT can inhibit ANDV infection at the level of virus entry. Pre-incubation of infectious virions with GRFT indicates that its antiviral effect is most likely due to direct interaction of GRFT with virion spike proteins. Hantavirus spikes contain tetramers of Gn-Gc heterodimers that cover the entire virion surface (Hepojoki et al., 2010; Huiskonen et al., 2010). Of the viral envelope glycoproteins, Gn harbors multiple *N*-linked glycosylation sites and has solvent-exposed positions (Shi et al., 2003), suggesting that it could be the main target of GRFT. Although not demonstrated experimentally, Gn is thought to bind receptors at the cell surface during entry (Mittler et al., 2019). It is likely that GRFT-glycan binding on the cell surface precludes the access of Gn to its receptor, although the possibility that GRFT binds to glycosylated cellular hantavirus receptors (Ethier et al., 2005; Mittler et al., 2019) cannot be ruled out. The demonstrated virucidal action of GRFT against HIV (Zeitlin et al., 2009) and Japanese encephalitis virus (Ishag et al., 2013) indicate the possibility of such happening in the case of hantaviruses. 3mGRFT consistently exhibited higher potency than GRFT in our experiments. The native homodimeric form of GRFT possesses 6 carbohydrate binding sites, whereas the synthetic trimer 3mGRFT has nine, suggesting a tighter interaction with glycans present on the surface of viral glycoproteins (Moulaei et al., 2015). Additionally, the inherent flexibility in conformation of 3mGRFT (Moulaei et al., 2015) may increase its avidity to a single Gn-Gc heterotetramer. Interestingly, GRFT did not potently inhibit ANDV post infection. This contrasts with other studies showing that GRFT affects multiple steps, including cell-cell fusion and viral spread in addition to entry (O'Keefe et al., 2009; Lusvarghi and Bewley, 2016; Lo et al., 2020). This difference could be due to several factors, including cell lines used and parameters chosen to measure viral replication. GRFT and 3mGRFT also inhibited SNV infection. Despite only sharing 77% amino acid identity, ANDV and SNV glycoproteins share conserved architecture of the Gn-Gc fold and *N*-glycosylation (Mittler et al., 2019; Warner et al., 2019). The finding that the *in vitro* potency of GRFT and 3mGRFT against ANDV and SNV is like that observed for other viruses (Lo et al., 2020) suggests that *in vivo* testing of GRFT and 3mGRFT against hantavirus infection is warranted.

## Data Availability Statement

All datasets generated for this study are included in the article/Supplementary Material.

## Author Contributions

PS-R conceived the study, performed experiments, analyzed results, and wrote manuscript. ML conceived the study and performed experiments. PC performed experiments. MF conceived the study and helped performed experiments. BO'K provided griffithsin and its synthetic trimer 3m-griffithsin. ML, MF, SN, JM, BO'K, and CS provided critical feedback for the study and reviewed the manuscript. All authors contributed to the article and approved the submitted version.

## Conflict of Interest

BO'K was a named inventor on a U.S. Government-owned patent on the protein griffithsin and its uses as an antiviral agent. The remaining authors declare that the research was conducted in the absence of any commercial or financial relationships that could be construed as a potential conflict of interest.

## Supplementary Material

Supplementary Figure 1 Griffithsin inhibits Andes virus replication in multiple cell types. Griffithsin (GRFT) inhibited Andes virus (ANDV) replication in a concentration-dependent manner. Huh7 cells or HT-1080 cells were treated for 1 h with varying concentrations of GRFT or 3mGRFT before infection with ANDV at a multiplicity of infection (MOI) of 0.1. At 72 h post infection, the cells were fixed, permeabilized, and stained with an antibody against Puumala virus nucleoprotein that is cross-reactive with ANDV nucleoprotein. Green, ANDV nucleoprotein; blue, cell nuclei; red, cell cytoplasm. **(A,C)** Dose-response curve showing the quantitation of ANDV-infected cells after GRFT **(A,B)** or 3mGRFT **(C,D)** treatment (% normalized to the vehicle-only control). Graphs represent the mean ± SD and are representative of 3 independent experiments, performed in quadruplicate.

Supplementary Figure 2 Dose response curve of cell viability of GRFT and 3mGRFT. Vero-E6 cells were treated with varying concentrations of GRFT or 3mGRFT. At 72 h post infection, cell viability was assayed using CellTiter-Glo assay reagent (Promega), with total luminescence measured using a Biotek HD1 synergy instrument. Luminescence levels (indicative of cellular ATP levels as a surrogate marker of cell viability) assayed in vehicle treated, uninfected cells were set as 100% viability. Dose response curves were fitted to the mean value of experimentally performed in triplicate.
