# Supplementary figures and images for: Hantavirus Infection Is Inhibited by Griffithsin in Cell Culture
Source: Front Cell Infect Microbiol. 2020 Nov 4;10:561502. doi: 10.3389/fcimb.2020.561502 (PMC7671970; doi:10.3389/fcimb.2020.561502)

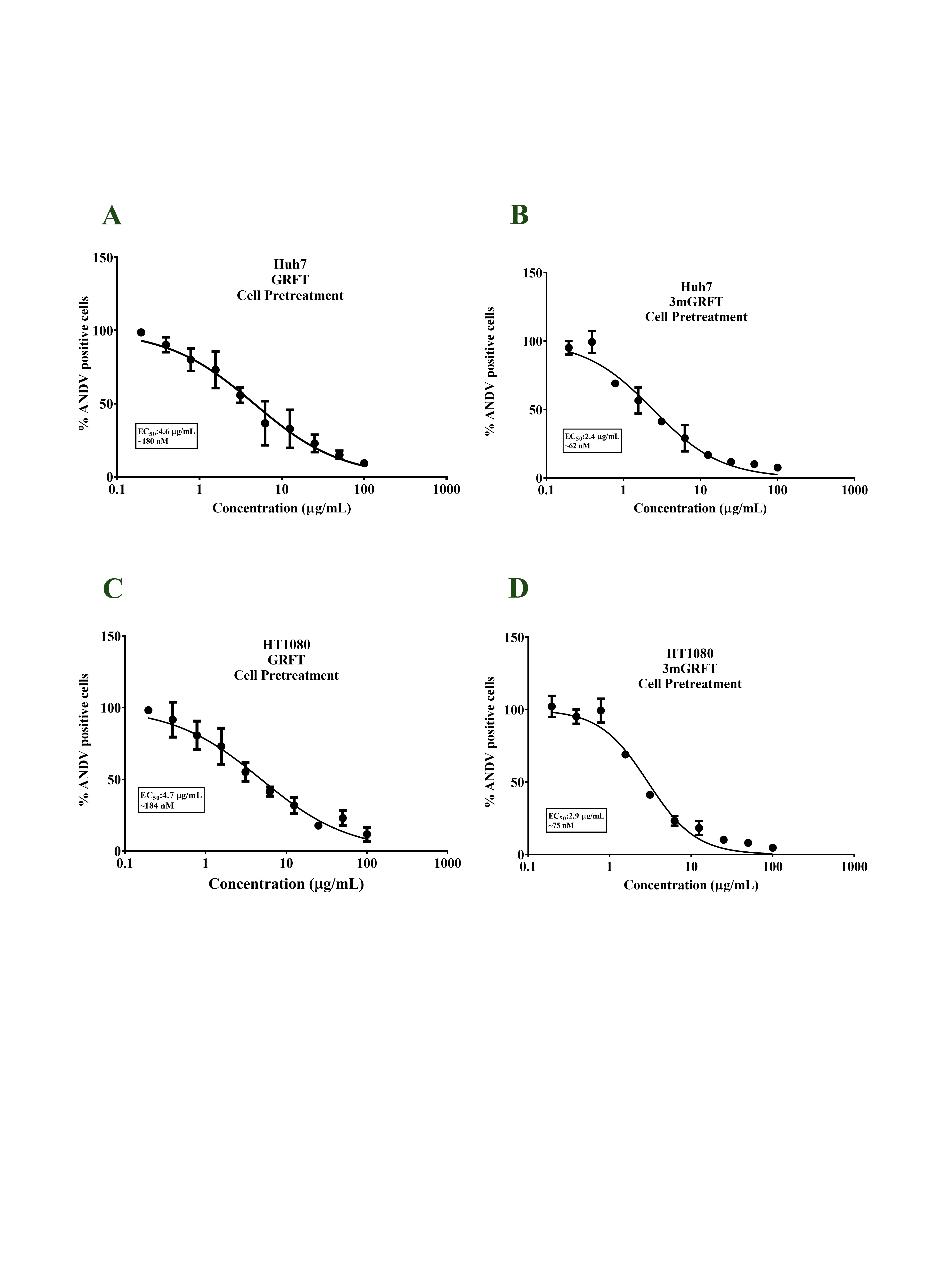

Supplement: Supplementary Figure 1 — Griffithsin inhibits Andes virus replication in multiple cell types. Griffithsin (GRFT) inhibited Andes virus (ANDV) replication in a concentration-dependent manner. Huh7 cells or HT-1080 cells were treated for 1 h with varying concentrations of GRFT or 3mGRFT before infection with ANDV at a multiplicity of infection (MOI) of 0.1. At 72 h post infection, the cells were fixed, permeabilized, and stained with an antibody against Puumala virus nucleoprotein that is cross-reactive with ANDV nucleoprotein. Green, ANDV nucleoprotein; blue, cell nuclei; red, cell cytoplasm. (A,C) Dose-response curve showing the quantitation of ANDV-infected cells after GRFT (A,B) or 3mGRFT (C,D) treatment (% normalized to the vehicle-only control). Graphs represent the mean ± SD and are representative of 3 independent experiments, performed in quadruplicate. [file Image_1.TIF]

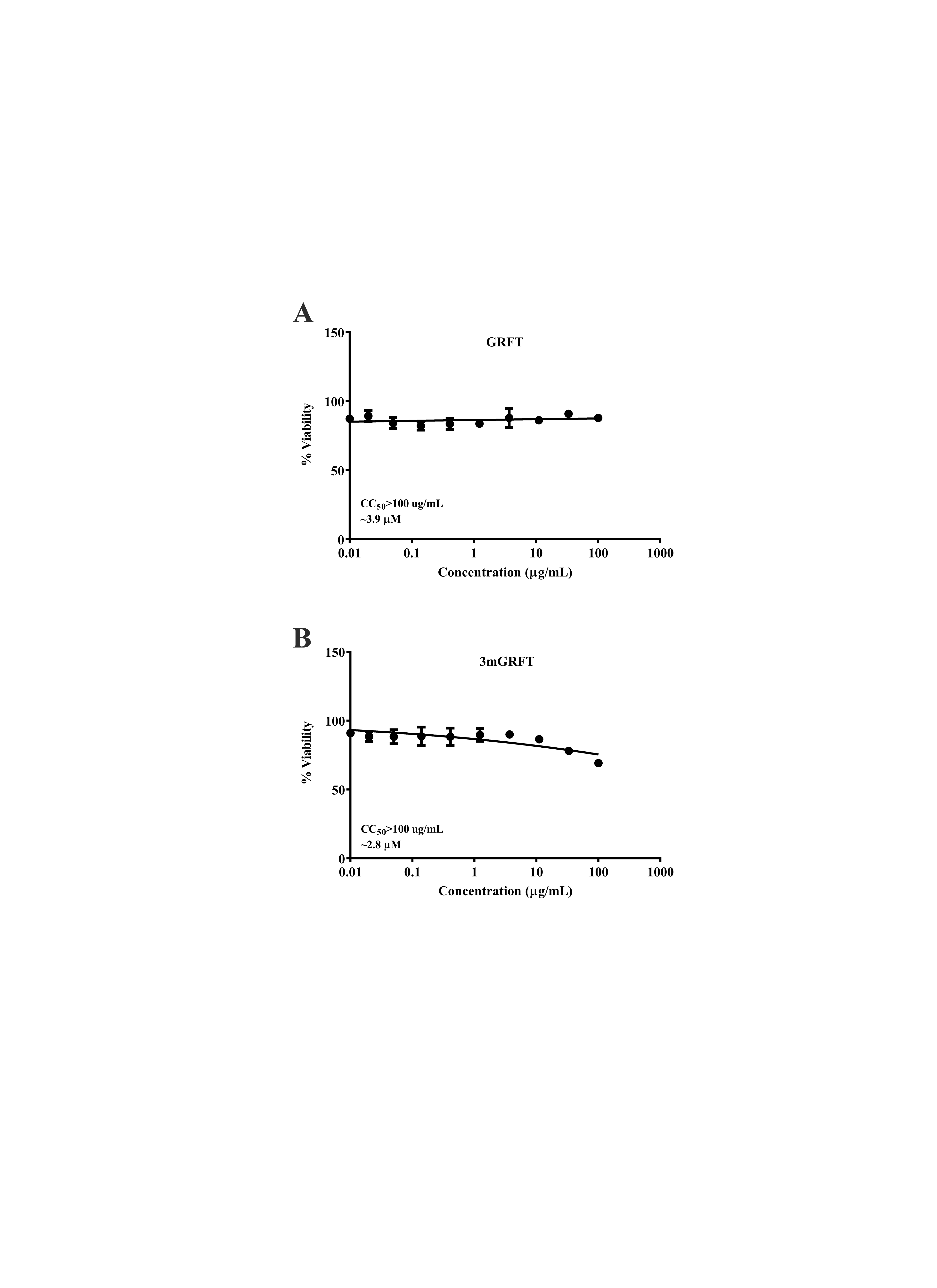

Supplement: Supplementary Figure 2 — Dose response curve of cell viability of GRFT and 3mGRFT. Vero-E6 cells were treated with varying concentrations of GRFT or 3mGRFT. At 72 h post infection, cell viability was assayed using CellTiter-Glo assay reagent (Promega), with total luminescence measured using a Biotek HD1 synergy instrument. Luminescence levels (indicative of cellular ATP levels as a surrogate marker of cell viability) assayed in vehicle treated, uninfected cells were set as 100% viability. Dose response curves were fitted to the mean value of experimentally performed in triplicate. [file Image_2.TIF]
